# Effect of Annealing Temperature on the Photoluminescence Blue Shift of Nitrogen-Doped Graphene Quantum Dots

**DOI:** 10.3390/ma18092094

**Published:** 2025-05-02

**Authors:** Xiaofen Xu, Jun Guo, Lu Han, Huimin Fan, Fei Tong

**Affiliations:** 1 School of Electronic Engineering, Huainan Normal University, Huainan 232038, China; 2 School of Materials Science and Engineering, Anhui University, Hefei 230601, China

**Keywords:** NGQDs, photoluminescence (PL), blue shift, mechanism

## Abstract

Nitrogen-doped graphene quantum dots (NGQDs) are made by heating a mixture of GQDs and ammonia using a thermochemical method. The optical properties of the samples were studied. Here, the role of the temperature used in the annealing process is investigated. It is found that with the increase in heating temperature, the sp^2^ fraction content continuously increases, and the photoluminescence (PL) blue shift continuously increases. The 550 nm peak of GQDs shifts from 550 nm to 513 nm NGQDs synthesized at 300 °C. The normalized PL intensity shows a significant blue shift in the emission peak of the NGQD samples compared to the GQDs. The peak position of the GQDs is 555 nm, while the peak positions of the NGQDs are 511 nm for NGQDs-250, 488 nm for NGQDs-300, and 480 nm for NGQDs-350. Using a simple thermochemical method, we can effectively dope N into GQDs, and it is evident from the electron energy loss spectra that N doping induces the emergence of a new energy level in the electronic structure, which alters the optical properties of NGQDs.

## 1. Introduction

Graphene quantum dots (GQDs) are graphene derivatives of nanometer size [1]; they have a non-zero bandgap and luminesce on excitation [2]. Chemically derived GQDs have shown very broad emission line width due to many kinds of chemical bonding with different energy levels, which significantly degrades the color purity and color tunability [3,4]. Amino functionalized materials have excellent and unique properties, such as good biocompatibility, high electrocatalytic activity, and tunable luminescent properties. These excellent properties enable NGQDs to be used in biomedical imaging, fuel cells, and environmental monitoring [5,6]. An electrochemiluminescence sensor based on nitrogen-doped GQDs (NGQDs), in which the NGQDs act as (ECL) emitters and sensitively remove catechol, has been reported [7]. After the emergence of coronavirus (COVID-19) in 2019, which has seriously affected people’s health, life, and work, many companies have used lipid nanoparticles (LNPs) or viral vectors to deliver genes related to SARS-CoV-2 spirochete virus proteins for vaccination. mRNA vaccines for COVID-19 have been successfully applied in gene therapy. However, the safety, stability, and efficiency of traditional transfection agents (e.g., LNPs and viral vectors) are still lacking and are not sufficient for gene therapy clinical applications [8]. Several researchers have synthesized NGQDs for transfection of genes such as messenger ribonucleic acids (mRNAs) and plasmid deoxyribonucleic acids (pDNAs). It has been shown that NGQDs (positively charged) can efficiently form electrostatic complexes with mRNA and pDNA (negatively charged) to successfully deliver genes to target cells, and the transfection efficiency of NGQDs is comparable to that of commercially available LNPs. In view of their excellent properties, NGQDs have great potential to become new universal gene transfer vectors beyond LNPs and viral vectors [8].

Currently there are many methods for the preparation of NGQDs, such as electrochemical [9], organic synthesis [10], and hydrothermal methods [11]. The primary approach to fabricate NGQDs was realized by either GO (graphene oxide) [12], CA (citric acid) [13], or other organics [14]. In view of the remarkable quantum-confinement and edge effect of quantum sized grapheme, chemical doping is an effective way to tailor their electronic characteristics and photoluminescence. However, there are still some issues that need to be addressed, such as the relatively narrow spectral coverage relative to other materials and the serious lack of qualitative control over optical properties [15]. Nitrogen doping can effectively improve the quantum yield of GQDs and modulate their luminescent properties. However, the nitrogen doping types of NGQDs obtained by different preparation methods are significantly different. At present, the preparation of NGQDs with controllable nitrogen doping types is still challenging, and the luminescence mechanism of NGQDs is still controversial. Therefore, there is still much room for research on the modulation of optical properties and luminescence mechanism of GQDs by nitrogen doping. Compared with the traditional doping method, the thermochemical reaction method of GQDs with ammonia does not require specific reaction conditions such as high pressure, and is simple, fast, green, pure, and easy to control. In this paper, NGQDs are synthesized by the thermochemical method, and their luminescence mechanism is investigated in detail.

In this paper, we report the synthesis of NGQDs by a thermochemical method using GQDs and NH_3_. The results showed that the sp^2^ fraction content and PL blue shift increased with increasing heating temperature. The 550 nm peak of GQDs shifted from 550 nm to a 513 nm peak of NGQDs synthesized at 300 °C. The normalized PL spectra showed that the spotlight intensity of the GQD and NGQD samples showed a significant blue shift. The peak positions of the GQDs were 555 nm, while the peak positions of the NGQDs were 511 nm for NGQDs-250, 488 nm for NGQDs-300, and 480 nm for NGQDs-350. From the experimental results, it was found that the NGQDs produced a new UV absorption peak at 289 nm, and from the energy level diagram of NGQDs, it is obvious that a new energy level (4.5 eV) appears between C (π*) and O (π*) due to the introduction of the N element.

## 2. Experimental

### 2.1. Materials

Vulcan XC-72 carbon black was purchased from Cabot Corporation (Billerica, MA, USA), nitric acid (15 mol/L) was bought from Alfa Aesar (Shanghai, China), and NH_3_ (99.999%) was bought from Guoxin Corporation (Nanning, China).

### 2.2. Production of NGQDs

**Figure 1:**
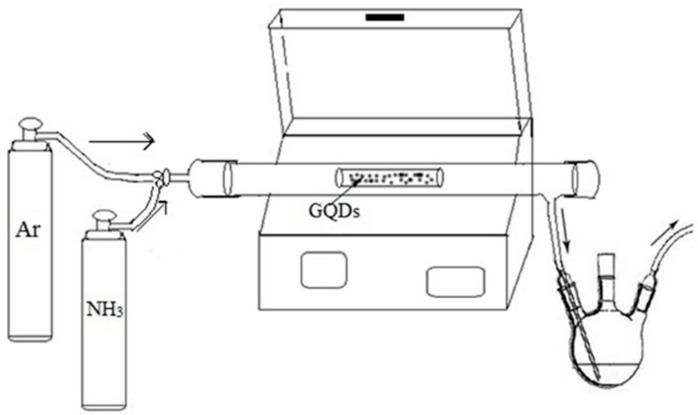
Schematic diagram of the experimental setup.

As mentioned earlier, GQDs were synthesized using Vulcan XC-72 carbon black and concentrated nitric acid (HNO_3_) oxidized by high temperature heating [16]. The specific experimental setup is shown in Figure 1. NGQDs were prepared using a homemade device in our laboratory. The specific operation is as follows: put the appropriate amount of GQDs into the quartz boat evenly dispersed, and then place the quartz boat in the quartz tube of the tube furnace. After closing the lid of the tube furnace, Ar and NH_3_ (about 99.99%) were passed into it at a rate of about 60 mL/min. It was then programmed to increase the temperature at a rate of 10 °C/min and to maintain a constant temperature for 1 h at a certain temperature. At the end of the process, the temperature was reduced at a rate of 10 °C/min until it reached room temperature. Finally, the NGQD samples were obtained as NGQDs-250, NGQDs-300, and NGQDs-350, where the numbers represent the heating temperature.

### 2.3. Characterizations

The Transmission Electron Microscopy (TEM) images were tested under the JEM-2100F (JEOL, Tokyo, Japan). The X-ray Photoelectron Spectroscopy (XPS) spectra were obtained with an ESCALAB 250Xi (Waltham, MA, USA). The infrared spectral results were measured by a Fourier transform infrared spectrometer (FT-IR) (Perkin-Elmer, Waltham, MA, USA). Raman spectra were recorded on a microscopic confocal laser Raman spectrometer (inVia, Renishaw, UK). The absorption spectra and PL spectra were measured by a UV-visible spectrometer (UV-2700, Shimadzu, Kyoto, Japan) and a fluorescence photometer (RF-5301PC, Shimadzu, Kyoto, Japan), respectively.

## 3. Results and Discussion

### 3.1. Microstructure Investigations

**Figure 2:**
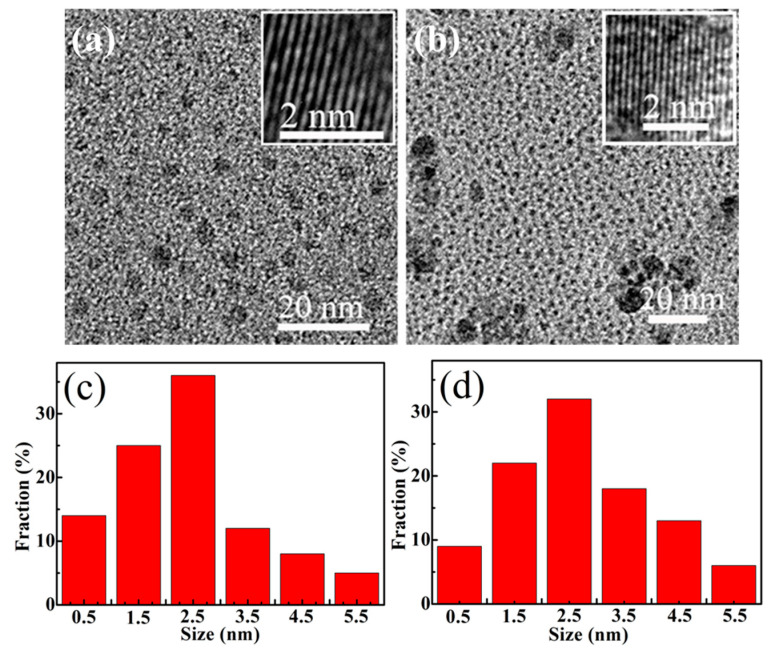
TEM images of the (**a**) GQDs and (**b**) NGQDs-300 (inset: HR-TEM images). The diameter distributions of (**c**) GQDs and (**d**) NGQDs-300.

As shown in the TEM images of the GQD samples (Figure 2a) and NGQD-300 samples (Figure 2b), the insets of Figure 2a,b show the high-resolution TEM (HR-TEM) images of GQDs and NGQDs-300, which indicate that both of the GQDs and NGQDs-300 have high crystallinity; Figure 2c,d are the corresponding diameter distributions of their samples. It can be seen that the particle size of the GQDs samples is uniform, while the NGQD-300 samples showed agglomeration. The reason for the agglomeration may be the preparation of the samples before the test, in which the sample solution was not ultrasonicated uniformly, or the operation was not standardized during the preparation of the samples, so that no better point was found during the test that would have a certain effect on the results of the sample morphology test. It is also speculated that the structure of the prepared NGQD-300 samples has been changed to a certain extent. Under the NH_3_ atmosphere, the high temperature heating will affect the internal structure of the GQDs to a certain extent, and the substantial decrease in its oxygen-containing functional groups will also have a certain impact on its measurement results. Meanwhile, when preparing the sample solution, it can be found that the solubility of the NGQD samples decreased with the increase in heating temperature, and the phenomenon of insolubility in deionized water appeared. From the high-resolution transmission electron micrographs, it can be seen that both GQDs and NGQDs-300 have a lattice stripe structure and belong to carbon materials, which indicates that nitrogen doping has no obvious effect on the lattice structure of GQDs. From the histograms of statistical distributions, the particle sizes of GQDs and NGQDs-300 are about 2.4 nm and 2.8 nm, respectively, which shows that there is no obvious change in the particle size, indicating that nitrogen doping has basically no effect on the morphology of GQDs. This experimental result is different from that previously reported [17] and is a powerful addition to explore the influencing factors of the fluorescence properties of NGQDs [2].

### 3.2. FTIR and Raman Investigations

**Figure 3:**
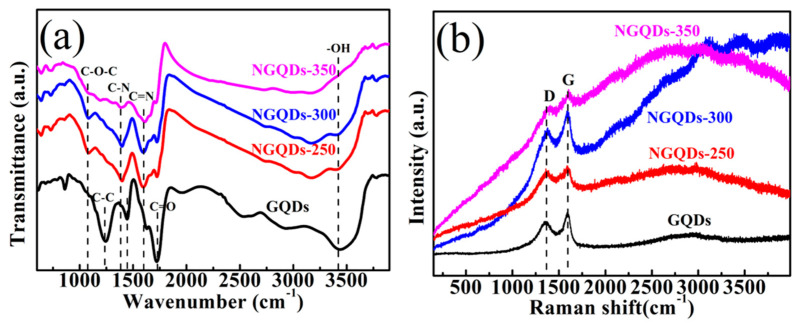
(**a**) FTIR and (**b**) Raman spectra of the GQD and NGQD samples.

Infrared spectroscopy allows us to know the chemical structure of the functional groups of the sample, which allows us to determine the type and structure of the compound, and the FTIR spectra of all the prepared samples are shown in Figure 3a. From this figure, the functional groups on the surface of GQD and NGQD samples can be analyzed and determined. The graphs show that all the samples show significant IR absorption peaks in the range of 3000~3500 cm^−1^, which is due to the stretching vibration of -OH [14,18]. The wave numbers at 1717, 1435 cm^−1^ are due to the telescoping vibration of C=O and COOH, respectively. The less intense absorption peaks at 1559 cm^−1^ and 1373 cm^−1^ are due to the stretching and bending of C=N and C-N, respectively, which indicates the presence of N in the NGQD samples, implying successful nitrogen doping of GQDs. The peak at 1220~1250 cm^−1^ is attributable to the C-OH, C-C telescoping vibration and bending vibration of -OH, and the peak at 1059 cm^−1^ is due to the stretching vibration of C-O-C bond [19,20,21,22]. It is obvious from the figure that the peak of vibration absorption for C=O is weakened after nitrogen doping in GQDs by the thermochemical method.

Raman spectroscopy [Figure 3b] can be used to qualitatively analyze the structure of GQD and NGQD samples to obtain information about their vibrations or rotation. The peak at ~1382 cm^−1^ (D) is due to the disorder of the sp^2^ hybridized carbon, and the peak at ~1617 cm^−1^ (G) corresponds to the E_2g_ mode of graphite, which is related to the vibration of the sp^2^-bonded carbon atoms in the 2D hexagonal lattice. The range of the D peak of NGQD samples is 1376–1398 cm^−1^ and the range of the G peak is 1586–1623 cm^−1^, however, the I_D_/I_G_ values of all the NGQD samples (0.88–0.95) are higher than that of the GQDs (0.87), and the experimental results are similar to those reported for nitrogen-doped graphite material [23]. In conclusion, the larger I_D_/I_G_ of the NGQD samples compared to the GQDs may indicate that the N is doped in the NGQDs. However, the NGQD samples exhibit a broader D band, suggesting that the intercalation of N atoms into the conjugated carbon backbone has led to somewhat disordered structures.

### 3.3. XPS Investigations

**Figure 4:**
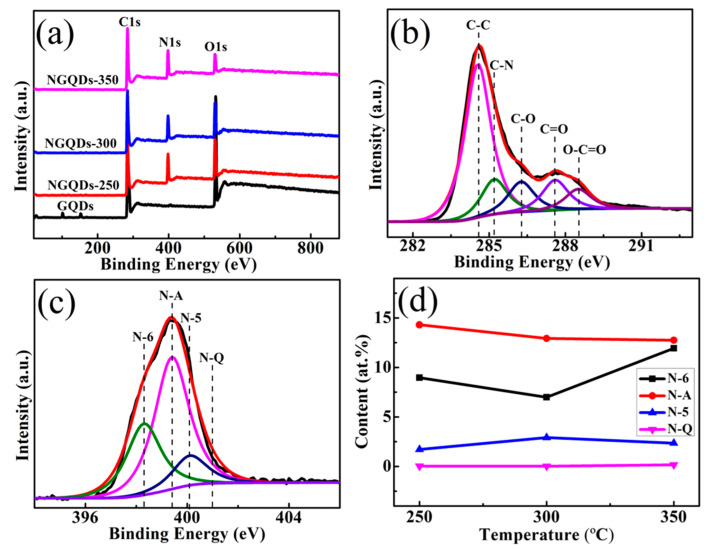
(**a**) The survey XPS spectra of GQD and NGQD samples. (**b**) C1s spectra of NGQDs-300. (**c**) N1s spectra of NGQDs-300. (**d**) Nitrogen-related components of the NGQD samples heated at different temperatures.

Moreover, as confirmed by XPS analyses, the prepared NGQD samples contained significant amounts of amino acids. As shown in Figure 4a, the full-range NGQD spectrum shows the binding energy peaks of C1s at 284.6 eV, N1s at 400.0 eV, and O1s at 532.0 eV. And NGQD samples show a clear N signal peak. Figure 4b shows the C1s XPS spectra of the NGQDs-300. These spectra consisted of peaks at 284.6, 285.2, 286.3, 287.6, and 288.5 eV, attributable to the C-C, C-N, C-O, C=O, and O-C=O groups, respectively [24,25]. The C-O bond may correspond to epoxy and tertiary alcohol functional groups on the basal plane, as well as phenol in the periphery. The C=O and O-C=O bonds indicate the presence of ketone and carboxylic groups in the grapheme periphery. As for the nitrogen functionalities, the N1s spectrum of NGQD samples [Figure 4c] comprised peaks corresponding to pyridine-like (N-6, 398.3 eV), amino-like (N-A, 399.4 eV), pyrrolidine-like (N-5, 400.1 eV), and graphite-like (N-Q, 401.7 eV) nitrogen atoms [16,26,27,28]. The content of the specific N types of the NGQDs was quantitatively showed in Figure 4d. One can see that with the increase in temperature of the NGQDs, the following phenomena will appear: (i) The contents of pyridine nitrogen (N-6) and pyrrole nitrogen (N-5) fluctuate up and down, while N-6 possesses the maximum value of 11.93 at.% for nitrogen-doped graphene quantum dots (NGQDs-350) heated at 350 °C, and N-5 for NGQDs-300 possesses the maximum value of 2.91 at.%; (ii) In all the samples, the N-Q stays in a relatively stable state and the contents are all relatively low; (iii) At different heating temperatures, all NGQD samples possessed high contents of N-A, indicating that N-A is the most predominant bonding type in NGQDs.

### 3.4. Optical and Photoelectrochemical Characteristics

**Figure 5:**
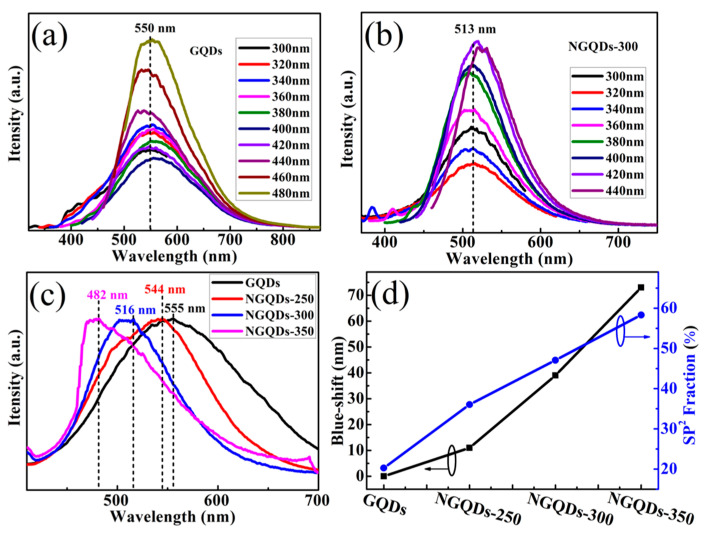
PL spectra of (**a**) GQDs and (**b**) NGQDs-300. (**c**) Normalized PL spectra of GQD and NGQD samples. (**d**) The dependence of the PL blue shift of the NGQD samples compared to GQDs on the value of sp^2^ fraction.

In order to investigate the effect of element N on the optical properties of GQD samples, photoluminescence (PL) spectra were performed. Figure 5a,b shows the PL spectra of GQD and NGQD-300 samples with different excitation wavelengths. It was found that as the excitation wavelength was varied (increasing from 300 nm to 480 nm); the emission peak of the GQDs was at 550 nm, thus showing an excitation-independent PL behavior (Figure 5a). However, when the excitation wavelength was increased from 300 nm to 440 nm, the emission peak position (513 nm) of NGQDs-300 was blue-shifted (Figure 5b), which was greatly different from the case of GQDs. In order to show the difference of the peak positions more clearly, the PL spectra were normalized so that the peak position was at 1.0. The normalized PL intensity of the GQD and NGQD samples shows a clear blue shift. When the excitation wavelength is 360 nm, the peak positions of GQDs, NGQDs-250, NGQDs-300, and NGQDs-350 are 555, 511, 488, and 480 nm, respectively (Figure 5c). Moreover, similarly to the NGQD materials reported, differences in the number of functionalized amino groups are responsible for the tunable photoluminescence, which are especially evident in the yellow-to-blue region [29]. It has been shown that protonation or deprotonation of functional groups can cause the PL emission of functionalized GQDs to shift with pH. PL offset is due to charge transfer between GQDs and functional groups, while the charge transfer can modulate the band gap of GQDs. The authors find that the results of calculations via density-functional theory (DFT) are in good agreement with their proposed mechanism for tuning the bandgap of GQDs via functionalization. In this paper, PL emission from functionalized GQDs appears red-shifted, unlike what we have reported [30]. In conclusion, the causes of photovoltaic shifts in NGQDs are complex and need to be further explored.

Many experimental results have demonstrated that a decrease in the size of GQDs leads to an increase in their bandgap [31,32]. For example, Peng et al. [33] performed acid treatment and chemical stripping of pitch-based carbon fibers to prepare GQDs of different sizes. The size of the GQDs is affected by the reaction temperature, which also controls the emission color and band gap. However, based on the experimental results, nitrogen doping was found to have little effect on the size of GQDs (see in Figure 2a–d), so it can be surmised that, unlike in the existing literature, the size of GQDs is not the cause of their photoluminescence changes in this paper. Therefore, we can speculate that the PL blue shift phenomenon of N-doped GQDs is independent of their size because of the quantum confinement effect of GQDs.

According to the literature, it is known that the peak area ratio of the sp^2^-bonded (C=C) carbon atoms can be calculated from the XPS test results to obtain the sp^2^ fraction. Discussion of the experimental results shows that the PL blue shift of the nitrogen-doped GQD samples is related to the value of the sp^2^ fraction. It is clear that in this paper, as shown in Figure 5d, the blue shift in the PL spectra of the NGQDs exhibits a similar trend to the increase in the sp^2^ carbon fraction as the annealing temperature increases. And the NGQDs-350 has the largest blue shift, of about 73 nm. Meanwhile, the sp^2^ fraction of NGQDs-350 has the maximum value of about 58.31%. During the high-temperature annealing process, the oxygen-containing functional groups in GQDs can be effectively removed, which leads to an increase in the proportion of sp^2^ carbon in NGQD samples [34,35]. Our experimental results further indicate that the NGQD photoluminescence blueshift is due to an increase in the sp^2^ fraction, from which we can determine the degree of photoluminescence blueshift by varying the value of the sp^2^ fraction of the GQD samples.

**Figure 6:**
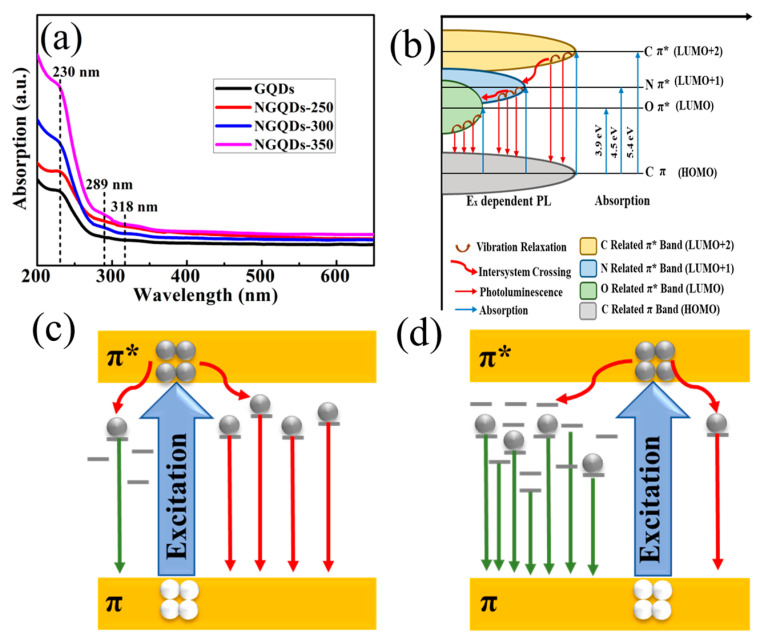
(**a**) UV-vis absorption spectra of the GQDs and NGQDs. (**b**) Schematic energy level diagram of NGQDs. Proposed PL emission mechanisms of (**c**) GQDs and (**d**) NGQDs.

At last, we tested the ultraviolet-visible (UV-Vis) absorption spectra of GQD and NGQD samples. The UV-vis absorption spectrum of the resultant NGQDs showed absorption bands at ca. 230 nm, 289 nm, and 318 nm [Figure 6a]. It has been reported in the literature that doping of carbon-based materials can modulate their properties, and the conduction type, electrical conductivity, and electrocatalytic activity of NGQDs can be modulated by doping with nitrogen [36,37,38]. It has been shown that the addition of N to GQDs can act to modulate the optical properties, resulting in a unique energy level structure [39]. Utilizing experimental data, we have deduced the energy level diagram for NGQDs [Figure 6b]. In the figure, we find three energy levels: C (π*), N (π*), and O (π*). The incorporation of nitrogen into the GQDs introduces a new energy level, which results in the emergence of an additional UV absorption peak. This peak is observed at a wavelength of 289 nm, corresponding to an energy of 4.5 electron volts (eV). This modification in the energy level structure enhances the optical properties of the NGQDs, indicating a shift in their electronic transitions and potentially broadening their applications in UV-responsive technologies. When a certain energy of light irradiates the NGQDs, the absorbed photons may cause electronic leaps of 5.4 eV (230 nm, π → π*, C=C), 4.5 eV (289 nm, π → π*, C=N), and 3.9 eV (318 nm, π → π*, C=O). The unique optical property of NGQDs, conferred by the nitrogen doping, manifests as three distinct absorption bands within the UV-visible spectrum. This characteristic spectral feature is a direct consequence of the nitrogen-induced energy level modifications, which facilitate the observation of these bands. The presence of these bands not only enriches the optical response of the NGQDs but also holds significant implications for their utility in various applications, such as sensors, photocatalysis, and optoelectronic devices, where tunable light absorption is crucial. Excited electrons are released in two main ways (Figure 6b). One is through direct recombination after vibrational relaxation, resulting in photoluminescence. The other is through intersystem crossing (C π* → N π*, N π* → O π*) followed by vibrational relaxation and finally radiative recombination. These two approaches give rise to PL with respect to excitation and PLE with respect to λ_Em_, leading to two dependencies with different slopes. It is clear that the “bridge effect” of N contributes to the intersystem crossover between NGQDs, making them a novel fluorescent material. Consequently, the varying electronegativity of nitrogen, upon doping, confers a distinctive structural arrangement to the GQDs. This arrangement engenders novel energy levels that significantly influence the material’s electrocatalytic performance, the nature of its charge carriers, and its electrical conductivity [40]. These excellent properties make NGQDs an important fluorescent material [39,41,42,43].

As reported, during the reduction of graphene oxide, its oxygen functional groups gradually decompose into CO or CO_2_ [34]. It is well known that the content of the sp^2^ structural domains of GQDs increases with a decrease in the oxygen functional group content (a decrease in the number of disordered induced states within the π-π* gap), which may lead to a blue shift in photoluminescence [35]. Figure 6c,d summarize the PL emission mechanisms of GQDs and NGQDs. It is recognized that the optoelectronic characteristics of carbon-based materials are primarily influenced by the π and π* energy levels associated with sp^2^ hybridized sites, which are positioned within the σ-σ* energy gap [44]. Furthermore, the PL emission observed in GQDs is predominantly attributed to sp^2^ hybridized carbon clusters that are either isolated within the sp^3^ carbon–oxygen matrix or associated with defects present in the GQDs [45,46,47]. As shown in Figure 4a, GQDs can be found to have a high content of oxygen-containing functional groups, implying that the components of the original GQDs are predominantly disorder-induced defect states within the numerous π-–π* gaps. It has been reported in the literature that (i) π-bonds are weaker compared to σ-bonds, thus forming lower energy; and (ii) Numerous localized states, arising from disorder, are present within the two-dimensional framework of GQDs. This framework is characterized by a significant proportion of carbon atoms that are distorted and bonded to oxygen-containing functional groups [46]. Due to the fact that the interactions among π states are heavily influenced by the projected dihedral angles, the localized states resulting from structural disorder could be situated at the periphery of the π-π* energy gap or potentially deeper within the gap. Consequently, optical transitions involving these localized states due to disorder might lead to the emission of a blue photoluminescence band at extended wavelengths (Figure 6c). The decrease in the number of disorder-induced states within the π-π* gap is accompanied by an increase in the number of cluster states formed by isolated sp^2^ domains. Consequently, the recombination of electrons and holes within the states of sp^2^ clusters leads to the manifestation of a violet hue in the photoluminescence at shorter wavelengths (Figure 6d) [46]. In other words, the quantity of minor sp^2^ structural domains could be augmented throughout the annealing procedure, resulting in the appearance of a change from blue to purple in the PL spectrum of the GQDs. It is shown that that the adjustable PL in the blue-to-yellow spectrum is attributed to variations in the quantity of amine groups that have undergone functionalization [29]. In the course of our work, NGQDs were prepared thermochemically by placing the GQD samples in a tube furnace and heating them under ammonia atmosphere. Similarly, the ammonia molecule breaks down into highly reactive ammonia radicals, which then bind to the GQDs via a radical addition reaction. It is considered that the nitrogen content in NGQDs varies irregularly with increasing annealing temperature. However, the oxygen content of NGQDs decreases with increasing annealing temperature, while the carbon content increases with increasing annealing temperature. Combined with Figure 5d, this indicates that heating under ammonia atmosphere is more favorable for the formation of sp^2^ structural domains with higher content than GQDs. Therefore, this further proves that the doping of nitrogen by the thermochemical method can effectively improve the optical blue shift of GQDs, and one can control the blue-shift level of NGQDs by adjusting the annealing temperature of the samples.

From the Raman, FTIR, XPS, PL, and UV-Vis analyses, it can be seen that the nitrogen content of the NGQDs is high, and the type of nitrogen shows an irregular change with the increase in the annealing temperature. From the TEM results, it is shown that doping does not significantly change the size of NGQDs, which means that the blue shift in the PL of NGQDs is primarily not influenced by their size, as the quantum confinement effect does not play a significant role. As the annealing temperature increases, the nitrogen content increases while the sp^2^ fraction increases continuously. From the analysis provided, it is inferred that the elevated nitrogen content, which is a consequence of the annealing temperature, is likely the principal factor responsible for the blue shift in the PL of NGQDs.

## 4. Conclusions

In this work, NGQDs were synthesized by heating GQDs in an ammonia atmosphere. It was found that the blue shift of NGQDs increased continuously with increasing temperature, and a maximum blue shift of about 73 nm could be obtained. Meanwhile, the content of the sp^2^ fraction also increased continuously with increasing temperature. Therefore, we speculate that nitrogen doping contributes to the photoluminescence blueshift of GQDs, and the excellent optical properties of NGQDs will lead to a wide range of applications in multicolor light-emitting devices, biological applications, and photovoltaics.

## Data Availability

The data presented in this study are available on request from the corresponding author due to (specify the reason for the restriction).
